# The first case of vesico-vaginal fistula in a patient with primary lymphoma of the bladder – a case report

**DOI:** 10.1186/1752-1947-1-105

**Published:** 2007-09-27

**Authors:** David A Evans, Andrew T Moore

**Affiliations:** 1 Kings Mill Hospital, Mansfield Road, Sutton-In-Ashfield, Nottinghamshire, NG17 4JT, UK

## Abstract

**Background:**

Primary lymphoma of the bladder is a rare condition with less than 100 cases reported in the literature.

**Case presentation:**

Here we present the case of a 64 year old woman with a 9 month history of haematuria, frequency, urinary incontinence and weight loss. Cystoscopy revealed a solid tumour throughout the whole bladder wall and a vesico-vaginal fistula. This was confirmed on CT scan which also showed no other organ involvement and bilateral hydronephrosis. Trans urethral biopsies taken at cystoscopy revealed Non-Hodgkin's Lymphoma. The patient had a nephrostomy inserted and is currently receiving a course of chemotherapy.

**Conclusion:**

We believe this is the first documented case of primary bladder lymphoma causing a fistula and therefore we suggest that lymphoma should be included in the differential for any patient with a fistula involving the bladder.

## Background

Non-epithelial tumours of the bladder are extremely rare. The most common amongst these are rhabdomyosarcomas in children and leiomyosarcomas in adults[1]. Primary lymphoma of the bladder is even less common accounting for 0.2% of all bladder neoplasms[2]. The most common site of primary extranodal malignant lymphoma is the stomach, followed by connective tissues and skin[3].

Primary lymphoma of the bladder was first described by Eve in 1885[4] and to date less than 100 cases have been reported in the literature[1,5]. Most lymphomas of the bladder are low grade Non-Hodgkin's lymphomas of the B-cell type[1,6]. Amongst the high grade subtypes of bladder lymphoma, diffuse large cell lymphoma is the commonest[7].

Considerable debate remains as to the origin of primary bladder lymphoma. This is largely because normal bladder microscopic sections fail to reveal routinely the presence of lymphoid follicles[8]. A popular theory is that lymphoma follows chronic cystitis, when there is an associated increase in the amount of lymphoid tissue found in the lamina propria[2,3,8,9]. A number of cases have been reported with absence of chronic cystitis and here it is postulated that the tumour may originate in the lymphoid tissue derived from the embryonic cloaca[8].

Lymphoma of the bladder is more common in women[2,3]. The most frequent presenting complaint is gross haematuria, occuring in 81.3% of reported cases. In 46.9%, a concomittent urinary tract infection is present. Dysuria and frequency are the only presenting symptoms in 18.7%[10]. Hydronephrosis is uncommon as the tumour does not typically involve the ureteric orifices. Involvement of the entire bladder wall is also a rare phenomonen[11-13].

## Case presentation

A 64 year old woman presented to our outpatients clinic with a 9 month history of painless haematuria, frequency and urinary incontinence. On further questioning she admitted to suffering from night sweats and 3 stones of weight loss over the past year. She had been repeatedly treated for urinary tract infections by her general practioner. There was no other past medical history apart from abdominal hysterectomy.

There was nothing abnormal to find on physical examination of the abdomen or other systems. The urea and electrolytes were normal as was the patient's peripheral blood film.

**Figure 1:**
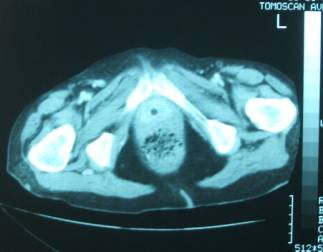
CT image showing thickened bladder wall with minimal lumen.

An abdominal ultrasound scan revealed bilateral hydronephrosis, a large solid pelvic mass with the dimensions 70 mm × 72 mm × 70 mm and no visible bladder despite attempts to fill with water. Cystoscopy showed solid tumor throughout the entire bladder with minimal lumen and a vaginal fistula. This was confirmed by a CT and showed a diffuse 30 mm circumferential thickening of bladder (see figure 1). There was no further lymphadenopathy apparent on CT.

Trans-urethral biopsies taken at cystoscopy revealed the tumour to be a diffuse Large and B Cell Non-Hodgkin's Lymphoma. A bone marrow biopsy revealed normal architecture and cell content.

**Figure 2:**
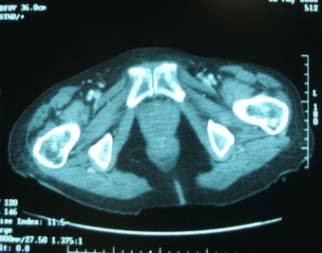
CT post chemotherapy showing relatively normal pelvic architecture.

The patient underwent insertion of a left nephrostomy tube under ultrasound guidance to improve renal function as a preparation for chemotherapy. She then received a course of R-CHOP (Cyclophosphamide, Doxyrubicin, Vincristine, Prednisolone and Ritoximab) chemotherapy. Four months later a repeat CT showed that changes in the region of the bladder were less marked (see figure 2). There was still right sided hydronephrosis and a nephrostomy on the left. The patient, symptomatically improved, remains under the care of the haematlogists.

## Conclusion

The diagnosis of primary bladder lymphoma was made after excluding a systemic haematological malignancy. Physical examination was normal; specifically there were no palpable lymph nodes and no organomegaly. No further lymphadenopathy was identified on abdominal or chest CT. The peripheral blood film and bone marrow biopsy were normal. This was in keeping with cases described in the literature[13-15].

This case demonstrates many of the commonly recognised features of this rare bladder tumour. Our patient was female, had a past medical history of chronic cystitis, presented initially with dysuria and haematuria and was ultimately shown to have a diffuse large cell lymphoma. It has been proposed that the higher incidence of urinary tract infection may be partly responsible for the higher frequency of bladder lymphoma in women. What is unique about this patient is that as far as we are aware this is the first documented case of a primary bladder lymphoma associated with a vesico-vaginal fistula.

We suggest that primary bladder lymphoma should be included in a differential for vesico-vaginal fistula. Conversely if a lymphoma is identified we suggest it would be pertinent to exclude a fistula.

## Competing interests

The author(s) declare that they have no competing interests.

## References

[B1] Mourad W Khalil S Radwi A Peracha A Ezzat A 'Primary T-Cell Lymphoma of the Urinary Bladder' The American Journal of Surgical Pathology 1998 22 3 373 377 10.1097/00000478-199803000-00014 9500781

[B2] Kuhara H Tamura Z Suchi T Hattori R Kinukawa T 'Primary malignant lymphoma of the urinary bladder. A case report' Acta Pathological Japonica 1990 40 10 764 769 10.1111/j.1440-1827.1990.tb01541.x 2291406

[B3] Freeman C Berg JW Cutler SJ 'Occurrence and prognosis of extranodal lymphomas' Cancer 1972 29 252 10.1002/1097-0142(197201)29:1<252::AID-CNCR2820290138>3.0.CO;2-# 5007387

[B4] Jacobs A Symington T 'Primary lymphosarcoma of urinary bladder' Brit J Urol 1953 25 119 125 13059372 10.1111/j.1464-410x.1953.tb09253.x

[B5] Wells W 'Primary low-grade B-cell lymphoma of mucosa-associated lymphoid tissue type arising in the urinary bladder' Arch Pathol Lab Med 2001 125 332 336 11231478 10.5858/2001-125-0332-PLGBCL

[B6] Fernandez Acenero MJ Martin Rodilla C Lopez Garcia-Asenjo J Coca Menchero S Sanz Esponera J 'Primary Malignant Lymphoma of the Bladder – Report of three cases' Path Res Pract 1996 192 160 163 8692717 10.1016/S0344-0338(96)80211-1

[B7] Melekos MD Matsouka P Fokaefs E Pantazakos A Repanti M 'Primary Non-Hodgkin's Lymphoma of the Urinary Bladder' Eur Urol 1992 21 85 88 1606990 10.1159/000474808

[B8] Aigen A Phillips M 'Primary Malignant Lymphoma of Urinary Bladder' Urology 1986 28 3 235 237 10.1016/0090-4295(86)90050-6 3750605

[B9] De Bruyne R Peters O Goossens A Braeckman J Denis LJ 'Primary IgG-lambda immunocytoma of the urinary bladder' European Journal of Surgical Oncology 1987 13 361 364 3114007

[B10] Santino AM Shumaker EJ Garces J 'Primary Malignant Lymphoma of the Bladder' The Journal of Urology 1970 103 310 313 5412748 10.1016/s0022-5347(17)61949-9

[B11] Arda K Ozdemir G Güneş Z Ozdemir H 'Primary malignant lymphoma of the bladder. A case report and review of the literature' International Urology and Nephrology 1997 29 3 319 322 10.1007/BF02550929 9285304

[B12] Bhansali SK Cameron KM ' Primary Malignant Lymphoma of the Bladder' British Journal of Urology 1960 32 440

[B13] Sharma A Singh AB 'Primary Lymphoma Of Urinary Bladder: Report Of An Unusual Case And Literature Review 'The Internet Journal of Urology' 2002 1 1

[B14] Guthman DA Malek RS Chapman WR Farrow GM 'Primary Malignant Lymphoma of the Bladder' The Journal of Urology 1990 144 1367 1369 2231929 10.1016/s0022-5347(17)39743-4

[B15] Pontius EE Nourse MH Paz L McCallum DC 'Primary Malignant Lymphomas of the Bladder' The Journal of Urology 1963 90 1 58 61 14047853 10.1016/S0022-5347(17)64360-X

